# Evidence and gap map of studies assessing the effectiveness of interventions for people with disabilities in low‐and middle‐income countries

**DOI:** 10.1002/cl2.1070

**Published:** 2020-01-09

**Authors:** Ashrita Saran, Howard White, Hannah Kuper

**Affiliations:** ^1^ Campbell Collaboration New Delhi India; ^2^ London School of Hygiene and Tropical Medicine (LSHTM) London UK

## Abstract

**Background:**

There are approximately 1 billion people in the world with some form of disability. This corresponds to approximately 15% of the world's population (World Report on Disability, 2011). The majority of people with disabilities (80%) live in low‐ and middle‐income countries (LMICs), where disability has been shown to disproportionately affect the most disadvantaged sector of the population. Decision makers need to know what works, and what does not, to best invest limited resources aimed at improving the well‐being of people with disabilities in LMICs. Systematic reviews and impact evaluations help answer this question. Improving the availability of existing evidence will help stakeholders to draw on current knowledge and to understand where new research investments can guide decision‐making on appropriate use of resources. Evidence and gap maps (EGMs) contribute by showing what evidence there is, and supporting the prioritization of global evidence synthesis needs and primary data collection.

**Objectives:**

The aim of this EGM is to identify, map and describe existing evidence of effectiveness studies and highlight gaps in evidence base for people with disabilities in LMICs. The map helps identify priority evidence gaps for systematic reviews and impact evaluations.

**Methods:**

The EGM included impact evaluation and systematic reviews assessing the effect of interventions for people with disabilities and their families/carers. These interventions were categorized across the five components of community‐based rehabilitation matrix; health, education, livelihood, social and empowerment. Included studies looked at outcomes such as, health, education, livelihoods, social inclusion and empowerment, and were published for LMICs from 2000 onwards until January 2018. The searches were conducted between February and March 2018. The EGM is presented as a matrix in which the rows are intervention categories (e.g., health) and subcategories (e.g., rehabilitation) and the column outcome domains (e.g., health) and subdomains (e.g., immunization). Each cell lists the studies for that intervention for those outcomes, with links to the available studies. Included studies were therefore mapped according to intervention and outcomes assessed and additional filters as region, population and study design were also coded. Critical appraisal of included systematic review was done using A Measurement Tool to Assess Systematic Reviews’ rating scale. We also quality‐rated the impact evaluation using a quality assessment tool based on various approaches to risk of bias assessment.

**Results:**

The map includes 166 studies, of which 59 are systematic reviews and 107 impact evaluation. The included impact evaluation are predominantly quasiexperimental studies (47%). The numbers of studies published each year have increased steadily from the year 2000, with the largest number published in 2017.The studies are unevenly distributed across intervention areas. Health is the most heavily populated area of the map. A total of 118 studies of the 166 studies concern health interventions. Education is next most heavily populated with 40 studies in the education intervention/outcome sector. There are relatively few studies for livelihoods and social, and virtually none for empowerment. The most frequent outcome measures are health‐related, including mental health and cognitive development (*n* = 93), rehabilitation (*n* = 32), mortality and morbidity (*n* = 23) and health check‐up (*n* = 15). Very few studies measured access to assistive devices, nutrition and immunization. Over half (*n* = 49) the impact evaluation come from upper‐middle income countries. There are also geographic gaps, most notably for low income countries (*n* = 9) and lower‐middle income countries (*n* = 34). There is a fair amount of evidence from South Asia (*n* = 73) and Sub‐Saharan Africa (*n* = 51). There is a significant gap with respect to study quality, especially with respect to impact evaluation. There appears to be a gap between the framing of the research, which is mostly within the medical model and not using the social model of disability.

**Conclusion:**

Investing in interventions to improve well‐being of people with disabilities will be critical to achieving the 2030 agenda for sustainable development goals. The EGM summarized here provides a starting point for researchers, decision makers and programme managers to access the available research evidence on the effectiveness of interventions for people with disabilities in LMICs in order to guide policy and programme activity, and encourage a more strategic, policy‐oriented approach to setting the future research agenda.

**Abbreviations**
3ie: International Initiative for Impact Evaluation
CBR: community‐based rehabilitation
CEDIL: Centre for Excellence for Development Impact and Learning
DFID: Department for International Development
EGM: evidence and gap maps
GDP: gross domestic product
LMIC: low‐ and middle‐income countries
RCT: randomised controlled trial
WHO: World Health Organisation
UNCRPD: United Nation Convention on the Rights of Persons with Disability
SDG: sustainable development goals

## 1 PLAIN LANGUAGE SUMMARY

### 1.1 The evidence for disability interventions is unevenly distributed by sector and geography, and much of it is of poor quality

There is a considerable body of evidence related to interventions for people with disabilities and their families in low‐ and middle‐income countries (LMICs), but it is unevenly distributed by sector and geography, and much of it is of low quality.

### 1.2 What is this evidence and gap map about?

There are approximately 1 billion people in the world with some form of disability—Approximately 15% of the world's population. The majority of people with disabilities (80%) live in LMICs, disproportionately affecting the most disadvantaged sector of the population.

Decision makers need to know what works, and what does not, to best invest limited resources to improve the well‐being of people with disabilities and their families in LMICs. This evidence and gap map (EGM) shows the available evidence from systematic reviews and impact evaluations.

**Table:** 

What is the aim of this EGM?
The aim of this EGM is to show all the available evidence from systematic reviews and impact evaluations of interventions to improve the welfare of people with disabilities and their families in low‐ and middle‐income countries (LMICs).

### 1.3 What studies are included?

The EGM includes impact evaluations and systematic reviews assessing the effect of interventions for people with disabilities and their families or carers. Included studies had to report an estimate of the quantitative impact of an intervention. The studies were categorised as to whether the intervention or outcomes focused on health, education, livelihood, social inclusion or empowerment.

The map includes 166 studies: 59 systematic reviews and 107 impact evaluations. The included impact evaluations are predominantly quasiexperimental studies.

### 1.4 What is the distribution of evidence?

The studies are unevenly distributed across intervention areas. Health is the most heavily populated area of the map: 118 studies of the 166 studies concern health interventions. Education is next most heavily populated (40 studies). There are relatively few studies for livelihoods and social, and virtually none for empowerment.

The most frequent outcome measures are health‐related, including mental health and cognitive development (*n* = 93), rehabilitation (*n* = 32), mortality and morbidity (*n* = 23) and health check‐up (*n* = 15). Very few studies measured access to assistive devices, nutrition or immunisation.

Over half (*n* = 49) the impact evaluations come from upper‐middle‐income countries. There are also geographic gaps, most notably for low‐income countries (n = 9). There is a fair amount of evidence from South Asia (n = 73) and Sub‐Saharan Africa (n = 51).

There is a significant lack of high‐quality studies, especially with respect to impact evaluation There also appears to be a gap in the framing of the research, which is mostly within the medical model and does not use the social model of disability. That is, the interventions mostly try to change characteristics of the person with a disability (e.g., improve social skills) rather than to address structures (e.g., readiness of schools to include people with learning disabilities).

### 1.5 What do the findings of this map mean?

The EGM summarised here provides a starting point for researchers, decision makers and programme managers to access the available research evidence on the effectiveness of interventions for people with disabilities in LMICs. This EGM is important in order to guide policy and programme activity, and encourage a more strategic, policy‐oriented approach to setting the future research agenda.

Whilst the evidence base is relatively large, it is unevenly distributed. There is a need for more studies in rights‐based approaches, livelihoods and empowerment. More studies are required from low‐income settings. And study quality needs to be improved for both impact evaluations and systematic reviews.

### 1.6 How up‐to‐date is this EGM?

The authors searched for studies published up to December 2018.

## 2 EXECUTIVE SUMMARY

### 2.1 Background

More than 1 billion people in the world have some form of disability. This corresponds to approxiamtely 15% of the world's population (World Health Organisation, 2011). The majority of people with disabilities (80%) live in LMICs where disability has been shown to disproportionately affect the most disadvantaged. In 2004, the World Bank estimated the global gross domestic product (GDP) loss due to disability to be between $1.71 trillion and $2.23 trillion annually, mainly because of the exclusion of people with disabilities from employment opportunities (Metts & Mondiale, 2004).

Although disability research in LMICs is growing, several important questions have not been adequately addressed. For example, what type of evidence is needed, and what are realistic expectations, to improve outcomes and inclusion for people with disabilities? This short report summarizes preliminary findings from an EGM commissioned by Department for International Development (DFID) under the Centre for Excellence for Development Impact and Learning (CEDIL) programme, and undertaken by the Campbell Collaboration and the International Centre for Evidence and Disability.

### 2.2 Objectives

The EGM presents studies of the effectiveness of interventions for people with disabilities and their families in LMICs across a range of outcome domains. Specifically, the objectives of the EGM were to:

a. Develop a clear framework of types of interventions and outcomes related to effectiveness of interventions for people with disabilities and their families in LMICs.
b. Map available systematic reviews and impact evaluation on the effectiveness of disability interventions in LMICs in this framework, with an overview provided in a summary report.
c. Provide database entries of included studies which summarize the intervention, context, study design and main findings.

### 2.3 What is an EGM?

An EGM is a presentation of the available, relevant evidence for a particular sector. Relevance is defined in relation to the scope of the map. This report is for a map of studies of the effectiveness of interventions to improve the welfare of people with disabilities and their families in LMICs. The map is a table or matrix which provides a visual presentation of the evidence. In the disability map the rows are intervention categories and the columns are indicator (outcome) categories. Both interventions and indicators have subcategories.

### 2.4 Search method

The search was conducted in three stages:

1. Populating the map based on a search of systematic reviews.
2. Populating the map based on search of Impact evaluation.
3. Populating the map based on grey literature search.

The search was carried out in February/March 2018 on: (a) academic databases, such as Medline and Web of Science; (b) international organization websites including DFID, (c) existing EGMs and (d) systematic review databases such as the Campbell Library. Only studies published since 2000 with a focus on one or more LMIC were eligible for inclusion. The search yielded over 46,000 hits, with over 35,000 hits coming from the search on OVID. One hundred sixty‐six studies were included in the final map after screening and coding.

### 2.5 Selection criteria

The target populations are people with disabilities and their families living in LMICs, based on the World Bank classification. According to the United Nation Convention on the Rights of Persons with Disabilities (UNCRPDs), people with disabilities include those who have long‐term physical, mental, intellectual or sensory impairments, which in interaction with various barriers may hinder their full and effective participation in society on an equal basis with others. The population sub‐groups of interest for this EGM include: women, vulnerable children (particularly children in care), conflict (conflict and postconflict settings), migrants and ethnic minority groups.

Studies with multiple populations are included in the map as long as they have a LMIC focus.

Reviews with a global focus are included if they did not have any search restriction excluding LMICs.

### 2.6 Screening, data extraction and quality appraisal

Title and abstract screening and the evidence classification were undertaken by two independent reviewers, and any discrepancies were resolved by a third reviewer. The studies that passed on to full text were screened against the eligibility criteria by two independent reviewers, and conflicts resolved by third reviewer. After screening, all studies were coded for a wide array of information and populated into the map.

The studies were coded by the intervention category and subcategory. The intervention categories are those from the WHO community‐based rehabilitation (CBR) guidelines (WHO, 2010): health, education, livelihood, social and empowerment. Advocacy and governance was added as a sixth category, given its importance to the DFID approach.

The coded information includes: bibliographic details for the study, the interventions from the framework that the study evaluates, the outcomes from the framework that the study measures and other relevant aspects such as population, region and countries. This coding was done by two independent reviewers and conflicts reconciled by a third reviewer. The quality of the included systematic reviews was assessed using A Measurement Tool to Assess Systematic Reviews (AMSTAR 2) and done independently by two reviewers. We also quality rated the impact evaluation (individual studies) based on the quality assessment tool for individual studies. This tool included six criteria (study design, sample size, attrition, definition of intervention, definition of outcome, baseline balance) that are appropriate for the assessment of quantitative impact evaluations

### 2.7 Results

The map includes 166 studies, of which 59 are systematic reviews and 107 impact evaluation. The included impact evaluation are predominantly quasiexperimental studies (47%). The numbers of studies have increased fairly steadily from the year 2000 with the largest number published in the year 2017.

The studies are unevenly distributed across intervention areas. Health is the most heavily populated area of the map. A total of 118 studies of the 166 studies concern health interventions. Education is next most heavily populated with 40 studies in the education intervention/outcome sector. There are relatively few studies for livelihoods and social, and virtually none for empowerment.

The most frequent outcome measures are health‐related, including mental health and cognitive development (*n* = 93), rehabilitation (*n* = 32), mortality and morbidity (*n* = 23) and health check‐up (*n* = 15). Very few studies measured access to assistive devices, nutrition or immunization.

Over half (*n* = 49) the impact evaluation come from upper‐middle income countries. There are important geographic gaps, most notably for low income countries (*n* = 9) and lower‐middle income countries (*n* = 34). There is a fair amount of evidence from South Asia (*n* = 73) and Sub‐Saharan Africa (*n* = 51). There is a significant gap with respect to study quality, especially with respect to impact evaluation. There appears to be a gap between the framing of the research, which appears to be mostly within the medical model (i.e., change at individual level) and not on the social model of disability (i.e., change at service or system level).

The majority of studies focus on people with physical impairments. There is a significant lack in studies focusing on people with visual or hearing impairment.

There is an important gap with respect to study quality, especially with respect to impact evaluation. Many of the included systematic reviews were assessed to have methodological limitations.

The findings from this EGM highlights a number of gaps, as mentioned above. Due to the strong concerns on the quality of reviews and impact evaluation, the evidence base needs to be strengthened on what works to improve the well‐being of people with disabilities and their families in LMICs. We identify the following implications for research:

1. More studies are needed to fill an important gap in measuring intervention for people with disability and incorporating considerations for equity, with increased focus on low income settings.
2. Ensuring that the funding and research agencies adopt best practice approach for conducting and reporting research to raise the quality of available data.

## 3 BACKGROUND

### 3.1 The problem, condition or issue

Disability is an umbrella term, covering impairments, activity limitations and participation restrictions. The Preamble to the UNCRPD acknowledges that disability is “an evolving concept”, but also stresses that “disability results from the interaction between persons with impairments and attitudinal and environmental barriers that hinder their full and effective participation in society on an equal basis with others” (United Nations General Assembly, 2006).

Impairments can relate to vision, hearing, physical, psychosocial and cognitive or other bodily functions. An impairment becomes disabling when individuals are prevented from participating fully in society because of social, political, economic, environmental or cultural factors. This definition of disability is in line with a biopsychosocial conceptualization of disability, recognising the importance of both the impairment and contextual factors in causing difficulties in participation. This definition informs the current EGM. This approach draws on the earlier, medical model focussed more on the importance of impairments, as well as the social model which concentrates on the role of society in the exclusion of people with impairments.

More than 1 billion people in the world have some form of disability. This corresponds to approximately 15% of the world's population (World Health Organisation, 2011). The majority of people with disabilities (80%) live in LMICs and disability disproportionately affects the most disadvantaged (Banks, Kuper, & Polack, 2017). People with disabilities are more likely to experience a range of exclusions, including from employment, education, healthcare access and social participation (World Health Organisation, 2011). As a consequence, people with disabilities are more likely to live in poverty, both because disability causes poverty, but also because people who are poor are more likely to become disabled (World Health Organisation, 2011). In addition to economic impact, employment serves many nonfinancial functions. For example, at the individual level, work provides a sense of purpose and belonging in society, leading to improved self‐esteem, greater autonomy and an enriched quality of life (Walsh & Tickle, 2013). More broadly, disability is linked to social exclusion and low levels of autonomy and sense of empowerment.

The link of disability and poverty is also borne at a global level, as evidenced by a large systematic review (Banks et al., 2017). In 2004, the World Bank estimated the global GDP loss due to disability to be between $1.71 trillion and $2.23 trillion annually (Metts & Mondiale, 2004). Turning to general disability, a World Bank study estimated that exclusion from the labour market results in a total loss of US$891 million/year in Bangladesh and that income losses among adult caregivers add an additional loss of US$234 million/year (World Bank, 2008).

People with disabilities are not a homogenous group, and include people with different ages, genders, impairment types and living in different settings, and this may influence the impact of disability. The systematic review showed that the link between poverty and disability is apparent for both males and females and regardless of poverty measure used (Banks et al., 2017). Poverty and disability were linked across impairment types, although a clearer link may have existed for people with mental conditions. Similarly, a study on link between poverty and disability found that people with mental illness face higher levels and intensity of poverty, partly as a result of stigma and prejudice (Trani & Loeb, 2012). There is some evidence that the relationship is strongest in countries with higher income level, that is, in upper–middle versus lower income countries. This means that as countries move out of poverty the people with disabilities are increasingly left behind. The review showed that the association of poverty and disability may be strongest in the working population age group. Similarly, another study that used internationally comparable data of 15 developing countries, found that people with disabilities aged 40 and above and people with multiple disabilities were more likely to be multidimensionally poor’ (Mitra, 2013).

A key argument in attaining welfare for people with disabilities is to equalize social and economic opportunities from both humanitarian and economic perspectives. From a humanitarian perspective, interventions are implemented to secure basic human rights for people with disabilities. From an economic perspective, programmes are expected to increase the human capital of people with disabilities, and thus enable them to reduce their dependence on income transfers and other forms of public support. This economic expectation addresses disability as a development issue. Research is now required to determine the most cost‐effective ways to overcome the above obstacles and develop policies and strategies that increase the economic contributions of people with disabilities (Metts & Mondiale, 2004).

Disability is also a human rights issue, as well as a development issue, and this is highlighted in a range of international documents, including the World Programme of Action Concerning Disabled People (WPA, 1982), the Convention on the Rights of the Child (CRC, 1989), the Standard Rules on the Equalisation of Opportunities for People with Disabilities (United Nation, 1994) and most importantly The United Nations Convention on the Rights of Persons with Disabilities (UNCRPD, 2006). The UNCRPD aims to “promote, protect and ensure the full and equal enjoyment of all human rights and fundamental freedoms by all persons with disabilities, and to promote respect for their inherent dignity”. It reflects the major shift in global understanding and responses towards disability, and emphasises that people with disabilities have the right for full inclusion.

Inclusive development includes and involves everyone, especially those who are marginalized and often discriminated against (UNDP, 2010). The justification for disability inclusive development is that unless people with disabilities are brought into mainstream it is impossible to break the cycle of poverty and discrimination. Attention to disability issues is now increasingly being seen in the policies and programmes of bilateral agencies like Department of International Development (DFID, 2000) either as part of inclusive new policies or in disability‐specific initiatives. Furthermore, disability is included as a specific focus within several of the sustainable development goals (SDGs). Although there is little data on the cost effectiveness of disability inclusive development, The Asian Development Bank maintains that the costs associated with including people with disabilities are far outweighed by the long‐term financial benefits to individuals, families and society (ADB, 2005; Banks & Polack, 2015).

### 3.2 The intervention

The “Twin‐Track approach” promotes integration of disability‐sensitive measures into the design, implementation, monitoring and evaluation of all development policies and programmes, called as “mainstreaming disability”, while simultaneously undertaking “targeted measures” such as disability‐specific policies, programmes and initiatives to ensure the inclusion and full enjoyment of human rights by persons with disabilities (UNDP, 2010). A twin‐track approach may be required to enable people with disabilities to contribute to creating opportunities, share in the benefits of development, and participate in decision‐making (DFID, 2000). The twin‐track approach aims to break this cycle between disability, poverty and exclusion, by both empowerment of individuals/families/organisations and by breaking down barriers in society, and is advocated for by many international donors (e.g., the World Bank, DFID, German Cooperation; the European Community [EC], the Finnish Cooperation) and non‐governmental organisation (NGOs).

The World Health Organisation's (WHO) CBR guidelines is based on this approach. CBR is a multisectoral, bottom‐up strategy which operates at the community level. While the UNCRPD provides the philosophy and policy of disability‐inclusive development, CBR is a practical strategy for its implementation (Mendis, Gunnel, Ann, & Einar, 1989). CBR activities are designed to meet the basic needs of people with disabilities, reduce poverty, and enable access to health, education, livelihood and social opportunities—all these activities fulfil the aims of the UNCRPD.

Therefore, the CBR will serve as a guiding framework for the EGM and the five pillars of CBR: health, education, livelihood, social and empowerment will form the intervention and outcome categories.

### 3.3 Why it is important to develop the EGM

Over the past decade, the academic literature on disability outcomes and effectiveness has grown substantially (Andresen, Lollar, & Meyers, 2000; Iemmi et al., 2015; Ramey et al., 2016). However, several important questions have not been adequately addressed. For example, what type of evidence is needed, and what are realistic expectations, for disability inclusive interventions? A lack of rigorous and comparable data on disability and evidence on programmes that work can impede understanding and action.

Understanding the numbers of people with disabilities and their circumstances can improve efforts to remove disabling barriers and provide services to allow people with disabilities to participate on an equal basis with others. Many efforts are currently underway to fill these knowledge gaps and generate internationally comparable data on the living situation and needs of people with disabilities. This is an important step towards persuading policy and programme decision makers that disability is an issue that needs urgent attention. However, it does not help them in determining which actions are required.

Knowledge production to influence policy and programme action takes place across several sectors (health, social welfare and education), focuses on various populations (different ages, ethnicities or with different needs), and involves rather diverse methodical approaches (e.g., systematic reviews, impact evaluation of different designs etc.). A mapping of the existing knowledge base is, therefore, required to provide a comprehensive overview of existing knowledge in this area, to improve the discoverability, and thereby the use, of that evidence. Furthermore, an EGM can show implementing agencies where there is no relevant information for their programmes and enable the purposeful and targeted commissioning of future research, tailored to the most eminent needs for knowledge and guidance. The EGM can also help to identify gaps to be filled by evidence synthesis where sufficient information is available within one subject area. This overview of the existing evidence is provided by the EGM presented in this study.

## 4 OBJECTIVES

### 4.1 Objectives

The EGM presents studies of the effectiveness of these interventions across a range of outcome domains. Specifically, the objectives of the map have been to:

i. Develop a clear framework of types of interventions and outcomes related to effectiveness of interventions for people with disabilities and their families in LMICs.
ii. Map available systematic reviews and impact evaluation on the effectiveness of disability interventions in LMICs in this framework, with an overview provided in a summary report.
iii. Provide database entries of included studies which summarize the intervention, context, study design and main findings.

## 5 METHODS

### 5.1 EGM: Definition and purpose

EGMs provide a visual overview of the availability of evidence for a particular sector–in this case “people with disabilities and their families”. The EGM consolidates what we know and do not know about “what works” by mapping out existing and ongoing systematic reviews and impact evaluations in this field; and provides a graphical display of areas with strong, medium, weak or nonexistent evidence on the effect of interventions or initiatives.

The EGM is presented in two dimensions: the rows list interventions and the columns list outcome domains. Each cell shows studies which contain evidence on that combination of intervention and outcomes. This EGM provides an overview of the existing systematic reviews and impact evaluations on the key outcome domains and interventions aimed to increase the welfare of people with disabilities in LMICs. EGMs show what studies are available. In accordance to the recommendations in the Campbell EGM Guidance document, maps do not summarize what the evidence says.

Impact evaluations are those intended to assess causal effects, also referred to as effectiveness studies. These studies are described in more detail in section below. Hence the map does not include the following sorts of studies: (a) prevalence studies of different impairments, (b) studies on the barriers and issues faced by people with disabilities, (c) process evaluations of interventions intended to benefit people with disabilities, and (d) ethnographic, participatory and other qualitative research or action research on people with disabilities. All of these studies are an important part of the body of research to understand and improve the lives of people with disabilities in LMICs, but they are not within the scope of this map.

The map has additional dimensions which capture study or intervention characteristics, such as study design, region, countries and population subgroup (which includes type of disability).

**Figure 1:**
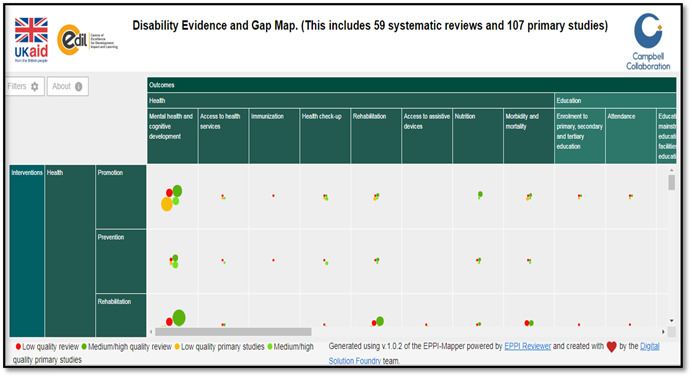
Snapshot of disability evidence and gap map

The online version of the map (Figure 1) is interactive so that users may click on entries to see a list of studies for any cell in the map. The map is constructed using software prepared by the EPPI Centre. The cells of the table contain a bubble whose size is proportional to the number of studies reporting that outcome for that intervention. There are separate bubbles for impact evaluation and systematic reviews, with the reviews further divided by study quality. The map includes a set of filters allowing evidence to be shown just for certain sub‐populations, such as specific regions or countries.

### 5.2 Types of evidence

The EGM of the effectiveness of interventions for people with disabilities shows the available evidence on the success of interventions to improve the lives of people with disabilities and their families in LMICs. Included studies adopt the International Classification of Functioning, Disability and Health, medical or social model conceptualization of disability.

The EGM includes systematic reviews of effects of interventions, as well as impact evaluations that used: (a) randomised experimental design, (b) rigorous quasiexperimental design, (c) natural experiments, (d) regression discontinuity, (e) propensity score matching, (f) difference in difference, (g) instrumental variables, (h) other matching designs or (i) single‐subject designs. Given the small number of studies in the map, the map also includes before versus after studies intended to address causal effects, though the absence of a comparison group means that we have low confidence in study findings from these studies.

The EGM includes both completed and on‐going studies. Ongoing studies are those which are in progress or the full review is not yet published. The reference for such studies is the study protocol.

### 5.3 Type of population

The target populations for this EGM are people with disabilities living in LMICs, based on the World Bank Classification (World Bank, 2017). We also included studies targeting parents/caregivers of people with disabilities. Other populations (e.g., teachers) may be targeted as a means for improving circumstances for people living with disabilities. For this map, we do not focus on the prevention of impairments.

People with disabilities include those who have long‐term physical, mental, intellectual or sensory impairments, which in interaction with various barriers may hinder their full and effective participation in society on an equal basis with others (UNCRPD, 2006).

For this map we will include following type of disabilities:

1. Physical: A physical impairment is the long‐term loss or impairment of part of a person's body function, resulting in a limitation of physical functioning, mobility, dexterity or stamina. It will include conditions as cerebral palsy, Spina Bifida, poliomyelitis, spinal cord injuries.
2. Visual: Visual impairment, also known as vision impairment or vision loss, is a decreased ability to see to a degree that causes problems in daily life. Conditions may include complete or partial loss of vision, due to conditions such as macular degeneration, retinal detachment and so on.
3. Hearing: Hearing impairment refers to partial or total inability to hear.
4. Intellectual: Also known as learning disability. This condition is characterized by significantly impaired intellectual and adaptive functioning which arises before the age of 18. This involves a permanent limitation in a person's ability to learn.
5. Mental: This category includes conditions such as Schizophrenia, Alzheimer's, bipolar disorders, psychosis.

If a paper includes mixture of disability types, that paper was coded for all types of disabilities as included. Similarly if the study included individual participant with multiple disabilities, again the study was coded for all those disabilities.

In recent years, the inclusion of traditionally underrepresented groups in research has received increasing attention, including racial and ethnic minorities, women, elderly individuals and children (Glickman et al., 2008). These groupings are relevant with respect to disability, as these characteristics may heighten vulnerability in the face of disability, and may also relate to a higher prevalence of disability.

Hence, the population sub‐groups of interest for this EGM include: women, vulnerable children (particularly children in care), conflict (conflict and postconflict settings), migrants and ethnic minority groups.

### 5.4 Types of interventions

The SDG guidelines highlight that implementing the SDGs should build upon existing international and national commitments and mechanisms, in order to generate an inclusive and global dialogue. The WHO's CBR recognizes CBR as a comprehensive and multisectoral strategy to equalize opportunities and include people with disabilities in all aspects of community life. Therefore, the CBR will serve as a guiding framework for the intervention and outcome categories as listed below, in order to realize the full inclusion and empowerment of persons with disabilities. We have added “Advocacy and Governance” as one of the components, as strong advocacy may be required to prevent and/or address abuse, neglect and exploitation that people with disabilities may experience (CBM, 2012).

The six main intervention categories are:

1. Health.
2. Education.
3. Livelihood.
4. Social.
5. Empowerment.
6. Advocacy and governance.

### 5.5 Types of outcome measures

The five main outcome categories are as mentioned below and they are plotted against the WHO's CBR indicators:

1. Health.
2. Education.
3. Livelihood.
4. Social.
5. Empowerment.

### 5.6 Types of settings

The EGM includes studies from LMICs. Studies with multiple populations are included in the map as long as they have a LMIC focus. Reviews with a global focus are included as eligible if they do not exclude countries from LMICs.

The World Bank region classification will be used as filters. There is also a filter for studies in conflict and postconflict settings.

### 5.7 Search methods and sources

The EGM is based on comprehensive search for impact evaluation and systematic reviews based on the framework of interventions and outcomes as outlined above.

The Campbell Collaboration policy brief for searching studies and information retrieval, informed the search strategy as presented below (Hammerstrøm, Wade, Jørgensen, & Hammerstrøm, 2010). In addition, information retrieval specialist John Eyers was consulted during the preparation of search strings, while several search retrieval specialists provided recommendations during the peer‐reviewing process of the study protocol. The lead author conducted the searches once the protocol had been peer‐reviewed and approved by Campbell Collaboration. The searches were conducted during the period February 19, 2018 to March 9, 2018.

At the end of the screening process, key journals were searched using key‐terms up to the end of January 2018. Studies in any language and from any country were included, provided the abstract was in English.

Searches were completed, as per protocol with a number of minor additions. In some cases the search string could be copied and pasted directly from the protocol, whilst other databases required the search string to be manually populated as recommended by Higgins and Green (2011), the search strategy is reported in Appendix B. Details of additional grey literature databases are included as recommended by Campbell Collaboration information retrieval specialists.

### 5.8 Electronic searches

The search was as comprehensive as possible, using (but not limited to) relevant systematic review database for first stage along with bibliographic databases (Appendix D), EGM databases, web‐based search engines, websites of specialist organisations, bibliographies of relevant reviews, and targeted calls for evidence using professional networks or public calls for submission of articles. Database for EGMs was also searched to identify any map and relevant populated studies. In addition, reference lists of the included reviews were reviewed and the authors contacted for information on other relevant sources. Citation searches were also performed (see Appendix B).

### 5.9 Searching other sources

We searched the following databases to identify unpublished reviews studies: Dissertation Abstracts, Conference Proceedings and Open Grey. We also searched a number of agency websites.

To identify ongoing studies, we searched ClinicalTrials.gov and WHO International Clinical Trials Registry Platform and CENTRAL Trials Register within the Cochrane Library for published trials.

### 5.10 Stakeholder engagement

An advisory group consisting of international experts in disability contributed to the preparation of the EGM by commenting on the EGM framework and advised on dissemination strategies. Members for this advisory panel are:

#### 5.10.1 Professor Tom Shakespeare

He is Professor of Disability Research, London School of Hygiene & Tropical Medicine. His primary research interests are in disability studies, medical sociology, and in social and ethical aspects of genetics. He has had a long involvement with the disabled people's movement in UK and internationally. In the context of disability arts, he has also been active in arts and culture, and was a member of Arts Council England from 2003 to 2008. During his 5 years at WHO, he helped produce and launch key reports such as the World Report on Disability (World Health Organisation, 2011) and International Perspectives on Spinal Cord Injury (World Heath Organisation & International Spinal Cord Society, 2013), and was responsible for the UN statement on forced, coerced and otherwise involuntary sterilization (World Health Organisation, 2014).

#### 5.10.2 Dr. David Olichini

He is the Head of Prevention and Health Unit, NCDs Technical Advisor, Humanity and Inclusion Federation. He leads the elaboration of the strategic plan for the Prevention and Health's Direction including definition of Key Performance Indicators, action plan and budget allocation for each of the sectors in the PHD, namely NCDs, Mental Health, Road safety and Sexual and reproductive health and right. He is the lead for various reports and publications on various aspects of disability.

#### 5.10.3 Professor G. V. S. Murthy

He is the Director, IIPH Hyderabad, India. His work revolves around improving global health and fostering international partnerships to improve health status of populations. He worked at WHO, Geneva on the Childhood Blindness Program and was a UNAIDS Consultant with National Aids Control Organisation (NACO) for 2 years, where he guided and monitored the first Behavioural Surveillance Survey undertaken by NACO and facilitated the development of the first Computerized Monitoring Information System for the National AIDS Control Program. He is an international expert on public health disability and has been engaged in generating evidence on health care access and health concerns of persons with disability and in developing innovative interventions to dismantle these barriers. He has undertaken research projects in India, Bhutan, Bangladesh, Pakistan, Nigeria, Nepal and Sri Lanka. Dr. Murthy is Technical Advisor on Disability to CBM South Asia, Technical Advisor (Research) for Mission for Vision. He is a member of the Queen Elizabeth Diamond Jubilee Trust's Scientific Advisory Committee, National Task Force on DR and ROP, Optometry Council of India, IAPB DR Technical Advisory Committee.

## 6 DIMENSIONS

### 6.1 Scope

The WHO's CBR programme recognizes CBR as a comprehensive and multisectoral strategy to equalize opportunities and include people with disabilities in all aspects of community life. CBR activities are designed to meet the basic needs of people with disabilities, reduce poverty, and enable access to health, education, livelihood and social opportunities—all these activities fulfil the aims of the UNCRPD. Therefore, the CBR will serve as a guiding framework and the five pillars of CBR: health, education, livelihood, social and empowerment will form the intervention and outcome categories. Clinical/pharmacological interventions to prevent or treat the primary impairment/health condition are beyond the scope of the map and hence such studies are excluded. We will be including studies that focus specifically on people with disabilities, as well as studies referring to interventions for families of people with disabilities.

### 6.2 Conceptual framework

**Figure 2:**
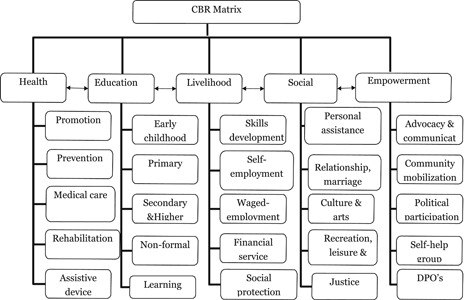
Community‐based rehabilitation matrix

The matrix (Figure 1) illustrates the different sectors, which can make up a CBR strategy for the welfare of people with disabilities. It consists of five key components, each divided into five key elements. The elements are subdivided into content headings. The matrix should not be seen as sequential, and all components will not be needed by every person with disabilities Figure 2.

### 6.3 Description of intervention/problem categories

The included interventions cover all main strategies to reduce disability related outcome as described in the CBR. The six main intervention categories are:

1. Health.
2. Education.
3. Livelihood.
4. Social.
5. Empowerment.
6. Advocacy and Governance (added—not part of CBR matrix).

### 6.4 Description of population/geographic location/outcome categories

The five main outcome categories are as mentioned below and they are plotted against the WHO's CBR indicators:

1. Health.
2. Education.
3. Livelihood.
4. Social.
5. Empowerment.

### 6.5 Description of population/geographic location

The EGM has two primary dimensions: interventions (rows) and outcomes (columns).

Additional dimensions are:

(1) Population subgroups of interest include: age group (under‐five, children, adolescent and elderly), women, vulnerable children (particularly children in care), conflict (conflict and postconflict settings), migrants and ethnic minority groups.
(2) Study designs: The EGM includes systematic reviews of effects of interventions and impact evaluations that used one of: (a) randomised experimental design, (b) quasiexperimental design (controlled before‐after and uncontrolled before‐after), (c) natural experiments, (d) regression discontinuity, (e) propensity score matching, (f) difference in difference, (g) instrumental variables and (i) single‐subject designs.
(3) World Bank region: South Asia, Sub‐Saharan Africa, East Asia and Pacific, Latin America and Caribbean, Middle East and North Africa, Europe and Central Asia.
(4) Type of impairment/disability: Physical impairment, visual impairment, mental impairment, hearing impairment, intellectual/learning impairment.
(5) Conflict‐affected regions.
(6) Economies: Low‐income economies, lower‐middle‐income economies, upper‐middle‐income economies, high‐income economies.

## 7 DATA COLLECTION AND ANALYSIS

### 7.1 Screening and study selection

An information specialist validated the detailed search strategy developed by the team covering a combination of academic databases, organisational websites and grey literature. Detailed search strategy is provided in the online protocol (link). All search results were imported in to EPPI reviewer for screening and coding.

The screening of studies in relation to inclusion/exclusion was undertaken in two stages. The first stage involved title and abstract; the second involved full text documents.

Three independent researchers were involved at each stage. The screening was carried out based on predefined eligibility criteria (Appendix A) by two independent reviewers and the third screener resolved the conflicts. Prior to data extraction and coding, the three independent reviewers met to discuss and pilot the extraction and coding procedures on a sample of abstracts.

Stage 1: Screen on Title and abstract

The screening was carried out based on predefined eligibility criteria (Appendix A) by two independent reviewers and the third screener resolved the conflicts. The conflict was resolved by third reviewer through group discussion with team. Title and abstracts which passed the first stage were retrieved in full text for a more comprehensive review.

Stage 2: Screen on full text

Full text documents were retrieved for all documents that passed stage one. Two reviewers independently evaluated all studies. Studies had to meet all of the inclusion/exclusion criteria set out previously in order to advance to full review.

### 7.2 Data extraction and management

Each included study was coded independently by two coders using the coding tool covering study characteristics, population, intervention, outcomes, region, countries and type of disability. The coding tool is added in the Appendix C.

### 7.3 Tools for assessing risk of bias/study quality of included reviews

Each study in the map has a rating for the quality of evidence.

For systematic reviews, we scored each study using the 16‐item checklist called AMSTAR 2 (“Assessing the Methodological Quality of Systematic Reviews”). The 16 items cover: (1) PICOS in inclusion criteria, (2) ex ante protocol, (3) rationale for included study designs, (4) comprehensive literature search, (5) duplicate screening, (6) duplicate data extraction, (7) list of excluded studies with justification, (8) adequate description of included studies, (9) adequate risk of bias assessment, (10) report sources of funding, (11) appropriate use of meta‐analysis, (12) risk of bias assessment for meta‐analysis, (13) allowance for risk of bias in discussing findings, (14) analysis of heterogeneity, (15) analysis of publication bias and (16) report conflicts of interest.

Items 2, 4, 7, 9, 11, 13 and 15 are termed “critical”. Study quality is rated high if there is no more than one noncritical weakness, and medium if there is no critical weakness but more than one non critical weakness. Studies with one or more critical weaknesses are rated low quality.

Impact evaluation: The quality assessment for the impact evaluations is based on existing approaches to risk of bias assessment. Many of the items in this assessment, such as study design and baseline balance, relate to possible sources of bias. Other items relate to clarity of reporting, especially of the intervention and outcomes. The assessment used the following criteria (see Tables 1, 2, 3):

1. Study design (potential confounders taken into account): Impact evaluations need either a well‐designed control group, preferably based on random assignment, or an estimation technique which controls for confounding and the associated possibility of selection bias.
2. Adequate sample size: Small samples generally mean that a study in underpowered, that is, there is a high risk of not finding an effect even if the intervention works.
3. Attrition (or loss to follow‐up) can be a major source of bias in studies, especially if these is differential attrition between the treatment and comparison group so that the two may no longer be balanced in preintervention characteristics. The US Institute of Education Sciences What Works Clearing House (Deke, Emily Sama‐Miller, & Alan Hershey, 2015) has developed standards for acceptable levels of attrition, in aggregate and the differential, which are applied here.
4. Clear definition of disability: For a study to be useful the study population must be clear, which means that the type and degree of disability should be clearly defined, preferably with reference to a widely‐used international standard (e.g., Washington Group questions).
5. Clear definition of outcome measures is needed in order to aid interpretation and reliability of findings and comparability with other studies. Studies should clearly state the outcomes being used with a definition and the basis on which they are measured, preferably with reference to a widely‐used international standard.
6. Baseline balance shows that the treatment and comparison groups are the same at baseline. Lack of balance can bias the results.

**Table 1:** Lists the intervention subcategories under each of these headings

CBR pillar (intervention category)	Component (intervention subcategory)	Examples
Health	Promotion	Parent/family training and education, inclusive health promotion campaigns, health care provider training
	Prevention	Introduction of specific intervention measures through better nutritional practices; improvement of health services, early detection and diagnosis; prenatal and postnatal care
	Medical care	Periodic health screening, access to routine healthcare
	Rehabilitation	Access to specialist care, such as physiotherapy, speech and language therapy, occupational therapy; cognitive stimulation, rehabilitation and training, activity therapy centres, supportive therapy, stress‐management interventions/psychosocial support, trauma informed therapy
	Assistive devices	Provision of appliances (orthoses, prostheses, hearing aids, etc.), devices such as day calendars with symbol pictures for people with cognitive impairment, communication boards and speech synthesizers for people with speech impairment
Education	Early childhood	Early intervention (e.g., play therapy), preschool/kindergarten provision
	Primary secondary and higher	Inclusive childhood education
		Provision of learning material and special equipment (Braille, audio cassettes, sign language, etc.), recruitment and training of specialized teachers, resource rooms, bypass intervention
	Nonformal	Faith‐based schools, home‐based learning, play groups
	Lifelong	Adult literacy programs, continuing education, life and survival skills
	Learning	
Livelihood	Skills development	Training opportunities for jobs, home‐based trainings, vocational training, training in mainstream institutions and community based trainings
	Self‐employment	Income generation program
	Financial services	Access to credit
	Waged employment	Quota legislation in jobs and participation in labour intensive public works programs
	Social protection	Social insurance schemes, social assistance intervention
Social	Relationship, marriage and family	Support in role as parents, protection from violence, building awareness in community of rights of people with disabilities to a family life
	Personal assistance	Provision, training and support of informal and formal personal assistance
	Culture and arts	Promoting use of art for social change like positive portrayal, silent theatres, complementary therapy in the form of art, music. Inclusive art education, diversity trainings, encouraging inclusion in mainstream cultural programmes, work with spiritual and religious leaders and groups
	Recreation, leisure and sports	Provision of adapted sports equipment, organization of inclusive sports events, linking people with disabilities to mainstream recreation and sporting clubs/associations, positive media coverage of disability recreation, using recreation
	Access to justice	Legal awareness, Identification of available resources like local leaders, legal centres, legal aid. Promoting legal rights and empowerment, inheritance right, community or legal aid centre
Empowerment	Communication	Improve access to information and communication resources; reduce communication barriers and improve representation for people with disabilities; Strengthen communication skills of CBR personnel
	Social mobilisation	Building trust and credibility within community, raise awareness in the community, motivate the community to participate, bringing stakeholder together, capacity building, celebrating achievements
	Political participation	Reservation of position in public and political institution, promoting political awareness and access to political process, disability awareness within political system
	Self‐help groups and disabled people's organisations (DPOs)	Creating joint resources like training material, community directories, advocating rights of persons with disability, partnership with existing self‐help groups
Advocacy and Governance		Establishment/reinforcement of a special education service in the Ministry of Education Establishment/Reinforcement of medical rehabilitation centres Legislative reforms: elimination of all forms of discrimination raising awareness on human rights through media appropriate budgetary allocation

Abbreviation: CBR, community‐based rehabilitation.

**Table 2:** List of outcome categories and subcategories

Outcome	WHO's community‐based rehabilitation (CBR) indicators
Health component	
Mental health and cognitive development	People with disabilities equally access mental health services and engage in activities needed to achieve the highest attainable standard of mental health services
Access to health services	People with disabilities equally access health services and engage in activities needed to achieve the highest attainable standard of health
	Percentage of people with disabilities and their families that have access to medical care
	People with disabilities feel they are respected and treated with dignity when receiving health services
Immunization	Percentage of people with disabilities who receive full immunization as recommended for their country by WHO
Health check‐up	People with disabilities know how to achieve good levels of health and participate in activities contributing to their health
	Percentage of children with disability who receive the recommended health check‐ups
Rehabilitation services	People with disabilities have access to, and use rehabilitation services
Access to assistive devices	People with disabilities have access to use, and know how to maintain appropriate assistive products in their daily life
Nutrition	People with disabilities have access to nutritional support to maintain a healthy diet
Morbidity and mortality	People with disabilities access and benefit from quality medical services appropriate to their life stage needs and priorities
Education	
Enrolment to primary, secondary and tertiary education	Policies and resources are conducive to quality education for people with disabilities and ensure smooth transitions through different stages of learning
	People with disabilities participate in and complete quality primary education in an enabling and supportive environment
	People with disabilities have resources and support to enrol and complete quality secondary and higher education in an enabling and supportive environment
	People with disabilities experience post school options on an equal basis with their peers
Attendance	People with disabilities have resources and support to enrol and complete quality secondary and higher education in an enabling and supportive environment
Education in mainstream education facilities/inclusive education	Percentage of people with disabilities who acquire education in mainstream education facilities
Social and life skill development	People with disabilities make use of youth or adult centered learning opportunities to improve their life skills and living conditions
Learning and achievement	People with disabilities experience equal opportunities to participate in learning opportunities that meet their needs and respect their rights
Access to educational services	People with disabilities participate in a variety of nonformal learning opportunities based on their needs and desires
	People with disabilities actively participate in early childhood developmental activities and play, either in a formal or informal environment
Livelihood	
Employment in formal and informal sector	People with disabilities have paid and decent work in the formal and informal sector on equal bases with others
	People with disabilities earn income through their own chosen economic activities
	People with disabilities acquire marketable skills on an equal basis with others through a range of inclusive training opportunities
Access to job market	People with disabilities have access to job markets on equal basis as others
Control over own money	People with disabilities have control over the money they earn
Access to financial services such as grants and loans	People with disabilities have access to grants, loans and other financial services on an equal basis with others
	People with disabilities participate in local saving and credit schemes
Poverty and out‐of‐pocket payment	Percentage of people with disabilities who are covered by social protection programs
Access to social protection programs	People with disabilities access formal and informal social protection measures they need
Participation in development of inclusive policies	Inclusive policies, practices and appropriate resources, defined with people with disabilities enable equal participation of women and men with disability in livelihood (training, finance, work opportunities and social protection)
Social	
Stigma and discrimination	Communities have increased awareness about disability, with a reduction in stigma and discrimination towards people with disabilities
Safety	People with disabilities feel safe in their family and community
Participation in mainstream recreational, leisure and sports activity	People with disabilities participate in inclusive or specific recreation, leisure and sports activities
Legal rights	All People with disabilities are recognized as equal citizens with legal capacity
Access to justice	People with disabilities access and use formal and informal mechanisms of justice
Participation in cultural and religious activity	People with disabilities participate in artistic, cultural or religious events in and outside their home as they choose
Interpersonal interaction and relationships	People with disabilities experience support of the community and their families to socialize and form age‐appropriate and respectful relationships
	Percentage of people with disabilities who feel respected in their decisions regarding personal relationships
Social identity and responsibilities	People with disabilities feel valued as community members and have a variety of social identities, roles and responsibilities
Empowerment	
Informed choices	People with disabilities make informed choices and decisions
Positions in public institutions and judiciary	People with disabilities participate in political processes on an equal basis with others
Voting rights	People with disabilities participate in political processes on an equal basis with others
Representation at community level	People with disabilities actively engage in and benefit from self‐help groups in the local communities, if they choose (inclusive or specific)
	Self‐help groups come together to form federations to harness collective energy and influence positive change
	People with disabilities living in different situations (rural or urban areas, poor or rich, refugees) feel they are adequately represented by DPO
Advocacy	People with disabilities effectively use communication skills and resources (including supportive decision making) to facilitate interactions and influence change
	People with disabilities play a catalyzing role in mobilizing key community stakeholders to create an enabling environment

Abbreviations: DPO, disabled people's organisation; WHO, World Health Organisation.

**Table 3:** Quality assessment of impact evaluation

Item		Point in time (where applicable)	Rating
1	Study design (potential confounders taken into account)	End of intervention	High confidence: Randomised controlled trial (RCT), regression discontinuity design, interrupted time series, instrumental variable
			Medium confidence: Difference in differences with matching, propensity score matching
			Low confidence: Other matching
2	Adequate sample size		High confidence: Sample size ≥100 or cluster ≥60
			Medium confidence: Sample size <100 or cluster <60
			Low confidence no power calculation or sample size <30 or cluster <30
3	Losses to follow up are presented and acceptable^a^	End of intervention	High: attrition within Institute of Education Science (IES) bounds^a^
			Medium: attrition close to IES bounds
			Low: attrition not reported or attrition outside IES bounds
			N/A for ex post studies
4	Intervention if clearly defined		High confidence: intervention clearly and fully described
			Medium confidence: brief description of intervention
			Low confidence: intervention named but not described, or not named
5	Outcome measures are clearly defined and reliable		High confidence: outcome measure clearly and fully described, preferably with reference to validation
			Medium confidence: brief description of outcome
			Low confidence: outcome named but not described
6	Baseline balance (not applicable for before versus after)		High confidence: RCT or baseline balance report and satisfactory (imbalance on 5 or less than 5 percent)
			Medium confidence: Imbalance between 5‐10 percent
			Low confidence: Baseline balance not reported, or reported and lack of balance on 10 or more than 10 percent
	Overall confidence in study findings	End of intervention	Lowest rating across items 1a, 4a, 6 and 7

^a^ https://homvee.acf.hhs.gov/HomVEE‐Attrition‐White_Paper‐7‐2015.pdf.

Overall study quality is the lowest rating awarded any one of the above six criteria.

## 8 RESULTS

### 8.1 Description of studies

#### 8.1.1 Results of the search

The search yielded over 46,000 hits, with over 35,000 hits coming from the search on OVID. Given the large number of hits, text mining on abstracts was used to narrow down the search results. EPPI reviewer uses machine‐learning algorithm (text‐mining) to prioritize the order in which references are presented for screening. The ranking of references continuously improves as screening progresses and more manual decisions are available from which the algorithm can learn. In EPPI Reviewer, citations are ranked in their order of relevance after choosing “starts priority screening”. This process fastens the screening process and left us with 9,842 hits, of which 237 were duplicates, leaving 9,606 studies for title and abstract screening. Of these, 547 studies were identified for full text screening and 100 were eligible for inclusion.

**Figure 3:**
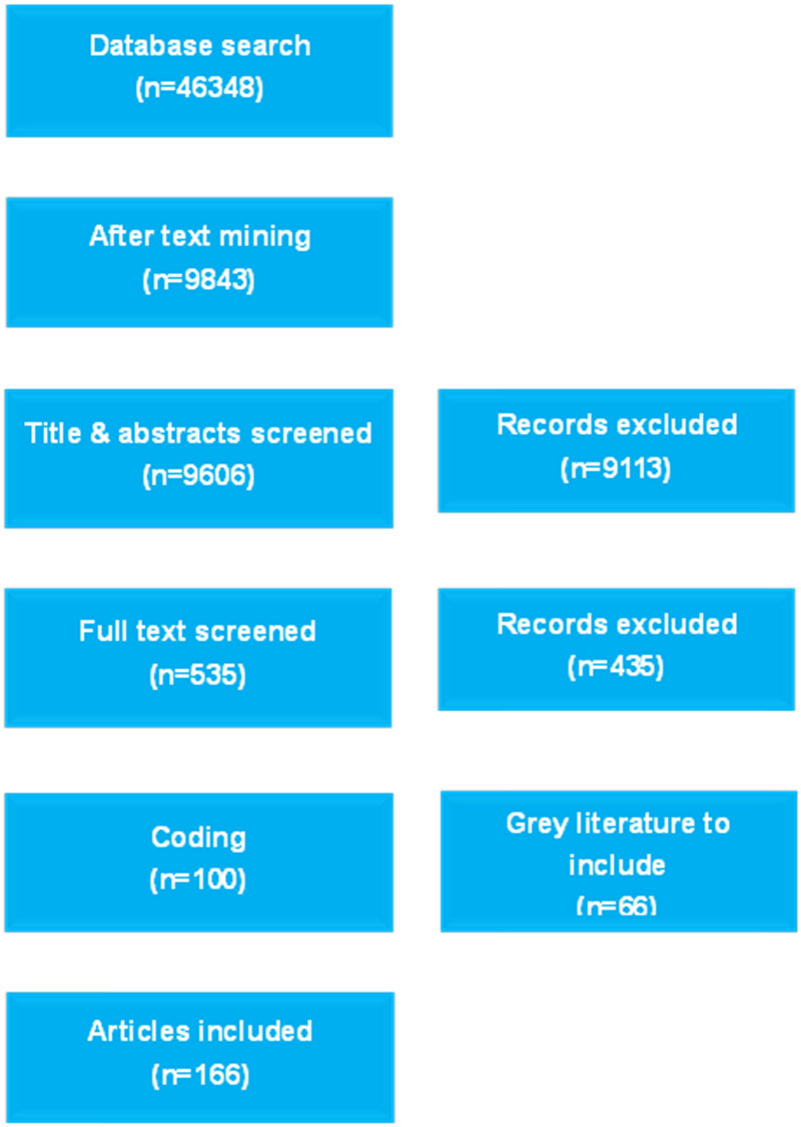
PRISMA for disability evidence and gap map

Phase 2 involved back screening included studies from systematic reviews and grey literature search. In Phase 2, an additional 35 studies were identified through back referencing. Grey literature search was performed and an additional 31 studies were included from grey literature. See the PRISMA flow chart in Figure 3.

As a result of this process, a total of 166 studies were included for coding. Of these, 59 are systematic reviews (see references for a list of included studies), and 107 impact evaluations. We then screened the included studies in the 59 systematic reviews to assess their eligibility for inclusion in the map.

### 8.2 Synthesis of included studies

Studies in the map, especially systematic reviews, may be coded under more than one intervention category or subcategory. This means that there are many more entries in the map then there are studies. The number of studies contained in the map is stated clearly at the top of the map.

### 8.3 Risk of bias in included reviews

**Figure 4:**
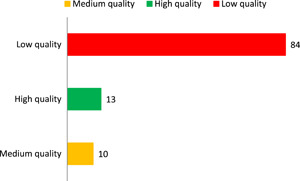
Number of impact evaluation by study quality

Figure 4 shows the results of the critical appraisal of the 107 included impact evaluations. Three quarters of the impact evaluations (78%) are rated as low confidence in study findings.

The high figure of low confidence is largely driven by concerns related to sample size and attrition. Seventy‐four percent of the impact evaluations had sample size <30. Attrition is not reported in half of the included studies.

### 8.4 Synthesis of included studies

#### 8.4.1 Publication of studies over time

**Figure 5:**
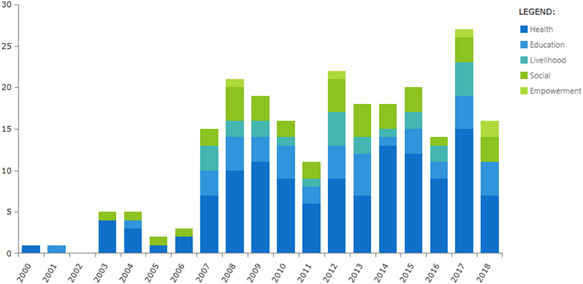
Trends in publication of studies by intervention

Figure 5 shows number of studies evaluating the effects of disability interventions published each year between 2000 and 2018. Since 2000 there has been a gradual increase in the number of studies from 1 to 21 new studies published in the year 2017. The search was conducted until end of January 2018 and hence it did not capture the studies published after January 2018. The majority of studies consistently focussed on health over the years.

**Figure 6:**
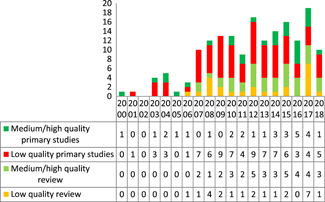
Quality of studies over the years

Figure 6 shows the quality trends of both impact evaluations and systematic reviews over the years. The proportional number of low quality impact evaluations has increased over the years as compared to medium/high quality impact evaluations. A high proportion of systematic reviews identified had methodological limitations and were of low quality.

### 8.5 By intervention categories

**Figure 7:**
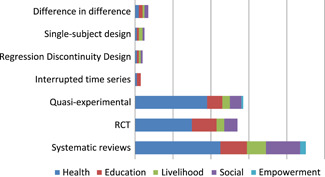
Number of studies by study design and intervention categories

Systematic reviews are concentrated in the health sector: 45 (80%) of reviews report effects of health interventions (Figure 7). Randomised controlled trials (RCTs) account for close to half of the impact evaluations (44 RCTs out of 107) being particularly prominent in health and education (30 and 13 studies, respectively), where some studies cover both sectors.

As mentioned above a single study may appear in more than one category. For example, Velema, Ebenso, and Fuzikawa (2008) review of rehabilitation programmes states that the interventions covered include “home visits by trained community workers who taught disabled persons skills to carry out activities of daily living, encouraged disabled children to go to school, helped find employment or an income generating activity, often involving vocational training and/or microcredit. Many programmes had a component of influencing community attitudes towards disabled persons”. This study is coded under each of health, education, livelihoods, empowerment and advocacy and governance intervention type.

### 8.6 By type of impairment

**Figure 8:**
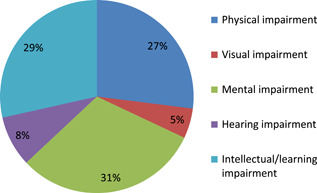
Number of studies relating to type of impairment

Nearly two‐thirds of the studies (60%) of the studies relate to interventions for people with mental or intellectual impairments, 27% to physical impairment, with a small number identified as relating to hearing and visual and hearing impairments (see Figure 8; recall that some studies are coded under more than one category).

### 8.7 By outcome domain

**Figure 9:**
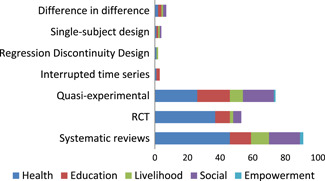
Number of studies by study design and outcome domain

Since the most common intervention category is health, it is unsurprising that the health‐related outcomes are reported in the largest number of studies (114 studies); see Figure 9. This is followed by education (46), social (46) and livelihoods (24 studies). Only 3 included studies report empowerment‐related outcomes. Systematic reviews are concentrated in the health sector: 46 (78%) of reviews report effects of health interventions (Figure 8). RCTs being particularly prominent in health sector (37) and considerably less than 10 RCTs in other sectors.

**Figure 10:**
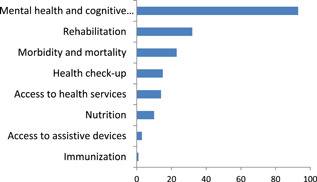
Number of studies by health outcomes

Within health, mental health and cognitive development account for the largest number of studies (93 studies) followed by rehabilitation (32) (Figure 10).

### 8.8 By region

**Figure 11:**
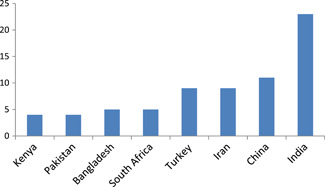
Countries with largest number of impact evaluations

**Figure 12:**
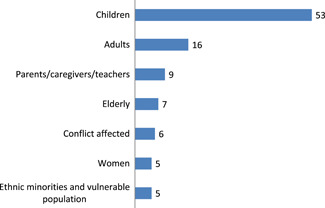
Completed Impact evaluations by population subgroup

**Figure 13:**
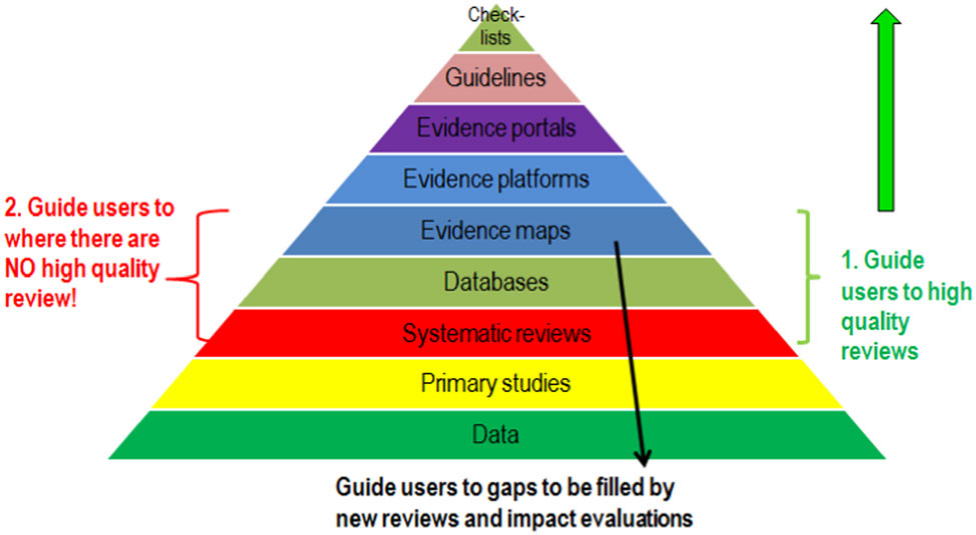
Evidence architecture. *Source*: White (2019)

Impact evaluations are unevenly distributed across World Bank region and countries (Table 4). Over half the impact evaluation come from four LMICs. These are concentrated in four countries: India (23), China (11), Iran and Turkey (9) studies each. South Asia is relatively well covered with studies from India (23), Bangladesh (5) and Pakistan (4) and as is East Asia on account of China (Figures 11, 12, 13).

**Table 4:** Countries in impact evaluation by income group

Low income		Lower‐middle income		Upper‐middle income	
*N* = 9		*N* = 50		*N* = 45	
Rwanda	1	Armenia	1	Turkey	9
Uganda	3	Indonesia	2	Iran	9
Ethiopia	3	Kenya	4	China	11
Eritrea	1	Bangladesh	5	Lebanon	1
Togo	1	Egypt, Arab Rep	3	Brazil	3
		India	23	South Africa	5
		Pakistan	4	Thailand	3
		Nigeria	3	Russia	1
		Vietnam	2	Peru	1
		Zambia	2	Malaysia	2
		Ukraine	1		

We included in the map all reviews in which studies from LMICs were eligible in searches. However, only 17 of the 59 included reviews actually include eligible studies. Of the other 44 reviews, 20 studies had only included studies from high‐income countries, 16 had LMIC studies which were not eligible for reasons of date or study design, 5 had no included studies and 2 are ongoing with results not yet reported.

Thirty‐eight impact evaluations concerned fragile and conflicted affected states (Table 5).

**Table 5:** Impact evaluation from fragile and conflicted‐affected states (*N* = 38)

High fragility	7
Pakistan	3
Nigeria	3
Eritrea	1
Moderate fragility	13
Lebanon	1
Iran	9
Egypt	3
Low fragility	11
Ethiopia	3
Bangladesh	5
Kenya	3
Neighbours	7
Zambia	1
Uganda	3
Thailand	3

### 8.9 By quality assessment

The systematic reviews were assessed using the AMSTAR tool described elsewhere in the document. Of the 59 reviews, 22 were assessed as low quality, 16 medium and 18 high quality, with the remaining three studies ongoing and, therefore, not yet scorable.

### 8.10 By population

Figure summarises the number of Impact evaluations by population. Fifty‐three impact evaluations focussed on children and sixteen on adults. Nine impact evaluations were identified that focused on interventions for parent/caregivers and teachers. There were limited impact evaluation on vulnerable groups and ethnic minorities.

### 8.11 Analysis by CBR pillars

#### 8.11.1 Health

The health quadrant of the EGM—which map the studies of the effects of health interventions on health outcomes—is the most heavily populated section of the map (Table 6). The total in Table 6 exceeds 166 (the total number of studies) as many studies are coded under more than one intervention and outcome.

**Table 6:** Aggregate map: Number of studies by intervention category and outcome (impact evaluation/reviews)

		Interventions					
		Health	Education	Livelihood	Social	Empowerment	Total
Outcomes	Health	58/44	9/10	6/5	11/17	1/0	161
	Education	10/9	21/11	2/3	6/8	1/1	67
	Livelihood	6/6	0/2	9/10	3/2	1/1	39
	Social	17/14	7/7	3/1	14/12	1/1	77
	Empowerment	1/2	0/0	0/0	1/1	1/2	7
	Total	166	66	38	71	10	351

Mental health is prominent in this quadrant: 93 studies report outcomes for mental health and cognitive development. Indeed, mental health dominates the map with the three largest bubbles being health intervention studies—medical care, rehabilitation and promotion—reporting a mental health outcome measure. Other heavily populated cells in the health domain are the rehabilitation outcomes from rehabilitation interventions, with an additional five reporting rehabilitation outcomes from health promotion interventions. Morbidity and mortality outcomes are also quite well represented with 25 studies, mainly from medical care and rehabilitation interventions.

Health interventions are generally the most heavily represented across nonhealth outcomes. Most notably there are 11 studies of rehabilitation interventions under the “social outcome” indicator interpersonal interaction and relationships.

#### 8.11.2 Education

Whilst 40 studies are classified in the intervention category and 46 studies have education‐related outcomes, closer analysis of these figures is needed to see the distribution of studies.

The studies classified under education very largely do not refer to participation of children with disabilities in formal education. The most commonly reported education outcome is “social and life skills development” (36 studies) with effects reported from health interventions (rehabilitation and promotion), as well as early child development, and nonformal education. On the intervention side, there are equal studies for nonformal education (18 studies) and primary/secondary (19 studies) and early child development (14).

#### 8.11.3 Livelihoods

Out of 22 included studies for livelihood interventions, 16 studies focused on skill development. On the outcome side 24 studies included livelihood outcomes. These two groups do not necessarily overlap; there are studies reporting the impact of livelihood interventions on health and livelihood outcomes, and there are studies analysing the impact of health interventions on livelihood outcomes. However, the studies included in the reviews are mostly of low quality and hence the relevant conclusions as made based on them can be undermined.^2017^, ^2011^

#### 8.11.4 Social and empowerment

Out of 35 studies included for social interventions 18 studies focus on relationship, marriage and family, followed by personal assistance interventions (16). On the outcome side 39 studies included interpersonal interaction and relationship. Evidence is sparse in the areas assessing the impact on stigma and discrimination (7), safety (4), participation in mainstream recreational, leisure and sports activity (2). Similarly very few studies are included for interventions as access to justice (4) and sports and recreation and leisure (6).

## 9 DISCUSSION AND GAPS IN EVIDENCE

Findings of this map are primarily helpful for researchers, policy makers and development practitioners that require evidence to inform policy and practice. National government and international partners can use it to identify existing evidence related to intervention of interest. Researcher and funders can identify areas suitable for evidence synthesis and move away from areas which may be saturated and also help explicitly identify gaps in knowledge.

EGMs are important building blocks in the evidence architecture and help in the following three ways:

1. Guide users to high quality reviews.
2. Guide users to where there are no high quality reviews.
3. Guide users to evidence gaps to be filled by new reviews and impact evaluations.

### 9.1 Areas of high quality reviews and impact evaluations

#### 9.1.1 Systematic reviews

Out of the 18 high quality review identified for this map, 13 (73%) are in health sector. A large proportion of these focuses on rehabilitation and health promotion.

Some high quality reviews were also identified in education (5) and social (5) pillars and may have some policy implications. Within education, high quality reviews were identified on early childhood Education and Non‐formal education. While only one high quality review was identified in the primary and secondary education.

A significant number of reviews were found to have methodological limitation, particularly in empowerment and livelihood sectors.

#### 9.1.2 Impact evaluation

Out of 13 high quality impact evaluations, 11 were identified in the health sector (85%). Even within health, there was unevenly distributed between subcategories; rehabilitation (6), medical care (4), promotion (3) and prevention (1). No high quality impact evaluations were identified on assistive devices.

High quality impact evaluations were scarce in other sectors and only one high quality impaction was identified in education, livelihood and social sector. No high quality impact evaluations are available on empowerment.

#### 9.1.3 Areas of major gaps in the evidence

We now summarise the important evidence gaps based on the analysis of included impact evaluations and systematic reviews in the map.

Many areas of the map are sparsely populated or unevenly distributed with evidence. The most evident gaps relate to empowerment and advocacy interventions and empowerment outcomes. For livelihood intervention, most of the areas are scarcely populated. There is only one study on self‐employment and limited studies on waged employment, financial services and social protection. For social interventions, sport, recreation and leisure and access to justice are scarcely populated.

Even where there are pockets of evidence, such as health and education, more studies are still needed. For instance in health, there are only few studies assessing effectiveness of assistive devices. Similarly in education, there are few studies on lifelong learning.

Following the same pattern, certain outcomes have received more attention than others. Health outcomes are certainly the most studies outcomes but gaps exist within this as well. Only three studies assessed access to assistive devices. Significant gaps exist in empowerment and livelihood outcomes. Within livelihood, outcome such as control over own money, poverty and out‐of‐pocket payments, access to social protection programs, participation in development of inclusive policies were least studied. Though social outcomes were fairly concentrated, limited studies were identified assessing stigma and discrimination, safety, participation in mainstream leisure and sports activity, legal rights, access to justice, participation in cultural and religious activities.

Most of the studies come from upper middle income countries, and even within this almost all the evidence comes from four countries, China, Turkey, Iran and South Africa. Similarly, in lower‐middle income countries most of the studies were undertaken in three countries:India (40), Pakistan (13) and Bangladesh (12).

There are also very few studies from low‐income countries, reflecting the relative neglect of many parts of Sub‐Saharan Africa such as Rwanda, South Sudan, Somalia, Congo, Burundi and so on. Studies were scarce for people with disabilities in many of these conflict affected regions.

There were also gaps by impairment type, with limited studies identified on people with visual and hearing impairment.

There is mostly low or medium confidence in study findings, and so another gap is the absence of high quality studies in the field. Reviews are of higher quality overall, though less than one‐third qualified as high quality and the studies they draw on tend to be of low quality.

We can draw on two Rapid Evidence Assessments undertaken from the EGM, and thereby go beyond the bounds of what reading the map can tell us (Kuper, Saran, & White, 2018; White, Saran, & Kuper, 2018). It is apparent from the reviews that the focus of studies is on fixing individuals, that is, the medical approach, for instance, focussing on improving social or learning skills for people with disabilities. Fewer studies focus on improving infrastructure or institutions, and therefore address social barriers to inclusion. Development agencies, including DFID, are stressing the biopsychosocial approach in their work, so the absence of evidence on what works to promote disability inclusion is a very striking gap. Future systematic reviews from the EGM will be able to provide more guidance for action for policy and programme decision‐makers.

#### 9.1.4 Limitations of the EGM

- The EGM provide a rich source of information on existing systematic reviews and impact evaluations relating to interventions to improve the lives of people with disabilities and their families in LMICs.
- The EGM followed comprehensive search using predefined eligibility criteria, yet inevitably there are limitations to our approach.
- Eligible studies were restricted to those published after 2000 up until the start of 2018, and published in English. Also searching the “grey” literature is challenging, and consequently some eligible studies may have been missed.
- Sometimes it was difficult for the reviewer team to categorize interventions, mainly between empowerment and livelihood, as there can be overlaps. The categorization for such interventions was done based on expert consultations and the information as available to mitigate this issue as far as possible.

## 10 AUTHORS’ CONCLUSIONS

The mapping exercise is a first step to identifying priority areas for systematic reviews and impact evaluations. We identify initial steps that can help advance research to promote the welfare and inclusion of people with disabilities. We strongly believe that the online interactive visualization, list of references, and summary of studies will facilitate access and use of research.

### 10.1 Implications for research, practice and/or policy

- The available high and medium quality systematic reviews in health sector may suggest some implications for policy. However, few of the studies are recent, and so they may need to be updated.
- Efforts are also needed to reach a consensus to identify priority areas for research with weak evidence synthesis by key funders and researchers in the field.
- More studies should be carried out given the relative lack of impact evaluation in many areas such as empowerment and livelihood. Impact evaluations will be more useful if they focus on more diverse set of outcomes and thereby fill multiple evidence gaps.
- More studies are needed to fill important gaps in equity and measuring interventions for vulnerable populations. This includes areas of gender, ethnic minorities and low‐income and conflict affected settings.
- The geographical base of evidence needs to be expanded as well. Most of the studies come from upper‐middle income countries and there is limited evidence from low income countries. Evidence need to be expanded in these countries.
- More studies are needed to generate evidence on all types of impairment, including visual and hearing impairments.
- Future research should also follow the best practice and improve reporting of intervention implementation in order to improve the quality of studies.
- Consideration needs to be given to improve quality of systematic reviews in terms of reporting and inclusion criteria or scope by adherence to standard guidelines as PRISMA.
- The future research agenda should explicitly consider the possibility for analyzing rights‐based approaches. A variety of evaluation designs might be appropriate: such as cluster randomization for community‐based approaches, and encouragement designs for national initiatives to promote inclusiveness.

## 11 INFORMATION ABOUT THIS EGM

### 11.1 EGM authors

#### 11.1.1 Lead EGM author

The lead author is the person who develops and co‐ordinates the EGM team, discusses and assigns roles for individual members of the team, liaises with the editorial base and takes responsibility for the on‐going updates of the EGM.

### 11.2 Contributions of authors

*Content expertise*:

Dr Hannah Kuper, Director of the International Centre for Evidence in Disability, a research group at LSHTM that works to expand the research and teaching activities of LSHTM in the field of global disability. Her main research interest is disability in low and middle income countries, with a particular focus on assessment of the prevalence of disability and impairments, including in children, and development of new methods in undertaking these surveys (e.g., use of mobile technologies), investigation of the health and rehabilitation needs of people with disabilities, and how these can be met in low resources settings and research on the relationship between poverty and disability, and the potential role of social protection in breaking this cycle. She has an undergraduate degree from Oxford University in Human Sciences and a doctorate from Harvard University in epidemiology. She has worked at LSHTM since 2002.

#### 11.2.1 Systematic review method expertise

All authors are experienced systematic reviewers, which means they are proficient in carrying out the various processes in an EGM, such as eligibility screening, quality assessment and coding.

*EGM methods expertise*:

All team members have previous experience in systematic review methodology, including search, data collection, statistical analysis, theory‐based synthesis, which mean they are proficient in carrying out the various processes in an EGM, such as search, eligibility screening, quality assessment and coding.

*Information retrieval expertise*:

All authors have previous experience in developing search strategies.

## CONFLICT OF INTEREST

No conflict of interests.

## PLANS FOR UPDATING THE EGM

Provided the availability of funding the EGM will be updated annually by lead author or co‐authors.

## DIFFERENCES BETWEEN PROTOCOL AND MAP

None.

## SOURCES OF SUPPORT

This EGM is supported by the UK Department of International Development (DFID) under its support for the Centre for Excellence for Development Impact and Learning (CEDIL). Dr Hannah Kuper is supported by the PENDA grant from DFID.

## APPENDIX A

**Table A1:** Description of methods used for inclusion and exclusion of studies

Selection criteria	Inclusion	Exclusion
Publication year	After 2000	Before 2000
Publication status	Completed and on‐going	None
Study design	The EGM will include systematic reviews of effects of interventions and effectiveness studies that used either: (a) randomised experimental design, or (b) rigorous quasiexperimental design, (c) natural experiments, (d) regression discontinuity, (e) propensity score matching, (f) difference in difference, (g) instrumental variables, (h) other matching design and (i) single subject design	Literature reviews, noneffectiveness studies, case studies and qualitative studies
Population	People with disability, and/or their family, their caregivers, their community living in low‐ and middle‐income countries	People with disabilities and/or their family, their caregivers, their community living in high‐income countries
	Disability is defined as impairments, activity limitations, and participation restrictions denoting the negative aspects of the interaction between an individual (with a health condition) and that individual's contextual factors (environmental and personal factors) (World Health Organisation, 2011)	
	For Impact evaluation we will include participants from low‐ and middle‐income countries only, as this was the original commitment of CBR (Helander, 1989)	
Interventions	We will include effectiveness studies.	All the clinical trials, interventions for reversible form of illness will be excluded.
	A CBR programme is formed by one or more activities in one or more of the five components (health, education, livelihood, social and empowerment). List of activities for each element of the five components are presented within the CBR guidelines under the section “Suggested activities”. The following activities are here given as examples: • Health: training PWD in the use of assistive devices; providing information to PWD and their family or their caregivers about time and location of activities for screening health conditions and impairments associated with disabilities • Education: providing education and training for families or caregivers of PWD; installing ramps in schools to make them accessible to PWD using wheelchairs • Livelihood: linking the jobseeker with disability to existing support services; advocating before relevant public and private agencies to ensure accessible housing for PWD • Social: converting institutions for PWD in rehabilitation centres; providing information to PWD about the sports opportunities available within the community • Empowerment: helping PWD running meetings of new self‐help group; involving disabled's people organizations in CBR planning, implementation, and monitoring	
		Interventions not focused on people with disabilities. We will also exclude studies that deals temporary or reversible form of disability for examples, maternal depression or back pain
Outcome	We will use the CBR framework for outcomes.	None
Quality	We will not restrict based on quality	None

## APPENDIX B

**Table B1:** List of databases

Indexes
International organizations
– ILO – DFID (including Research for Development [R4D]) – UNESCO – WHO – Disability Programme of the United Nations Economic and Social Commission for Asia and the Pacific (UNSCAP) – United States Agency for International Development (USAID)
Evidence and gap map databases
– International Initiative for Impact Evaluation (3ie) Evidence and gap map repository – Swedish Agency For Health Technology Assessment and Assessment of Social Services – Collaboration for Environmental Evidence – Global Evidence Mapping Initiative – Evidence based Synthesis Program (Department of Veteran Affairs) – Cochrane – Evidence based policing matrix – EPPI Centre Evaluation Database of Education Research
Systematic review databases
– Cochrane – Campbell – 3ie Systematic Review Database – Research for Development – Epistemonikos
Academic databases
– Econlit – The National Bureau of Economic Research (NBER) – Social Science Research Network (SSRN) – International Bibliography of Social Sciences (IBSS) – Applied Social Sciences Index and Abstracts (ASSIA) – Embase – PsycINFO – MEDLINE – WHO's Global Health Library – CABI's Global Health – ERIC – CINHAL – SCOPUS – Web of Science
– Other websites
– Humanity and Inclusion (HI) http://www.hi‐us.org/publications – CBM https://www.cbm.org/Publications‐252011.php – Plan international https://plan‐international.org/publications – PAHO https://www.paho.org/hq/index.php?lang=en – UNICEF www.unicef.org/ – UNICEF Innocenti Research Centre www.unicef‐irc.org/ – UN Women http://www.unwomen.org/en/digital‐library/publications – UNESCO http://www.unesco.org/library/e‐resDatabases1.html – United Nations Population Fund http://digitallibrary.un.org/record/703986 – UN Economic and Social Council www.un.org/en/ecosoc/ – IRC https://www.rescue.org/reports‐and‐resources – IFRC https://www.ifrc.org/en/publications‐and‐reports/ – CARE www.care.org – Indian Citation Index (ICI) http://www.indiancitationindex.com/ – Save the Children www.savethechildren.org – British Library for Development Studies http://blds.ids.ac.uk/ – ELDIS http://www.eldis.org/ – Essential Health Links http://www.healthnet.org/essential‐health‐links – Global Health and Global Health Archive http://www.cabi.org/datapage.asp?iDocID=169 – African developmental bank https://www.afdb.org/en/knowledge/african‐development‐institute/information‐and‐library‐services/ – Young Lives https://www.younglife.org/ForEveryKid/YoungLives/Pages/default.aspx – Association for the Development of Africa http://www.adeanet.org/en/knowledge‐and‐resources – Médians Sans Frontières www.msf.org.uk/ and http://fieldresearch.msf.org/msf/ – Action Aid http://www.actionaid.org/publications – ILO https://www.ilo.org/global/publications/lang‐‐en/index.htm – DFID (including Research for Development (R4D) https://www.gov.uk/dfid‐research‐outputs – Disability Programme of the United Nations Economic and Social Commission for Asia and the Pacific (UNSCAP) https://www.unescap.org/our‐work/social‐development/disability – United States Agency for International Development (USAID) https://www.usaid.gov/kyrgyz‐republic/key‐documents – World Vision www.worldvision.org.uk/ – Department for International Development www.dfid.gov.uk/ – World Food Programme https://www.wfp.org/evaluation/list (Evaluations) – Valid International www.validinternational.org/ – Concern Worldwide www.concern.net/ – International Red Cross/Red Crescent www.ifrc.org/

## APPENDIX C

**Table C1:** Coding tool

Category		Answer
Descriptive information	Title	Open answer
	Author citation	Open answer
	Publication date	Open answer
	URL	Open answer
	Volume no	Open answer
	Issue no	Open answer
Geographical information	World Bank region	– South Asia – Sub‐Saharan Africa – East Asia and Pacific – Europe and Central Asia – Latin America and Caribbean – Middle East and North Africa – North America
	Country	– Low income – Lower Middle income – Upper Middle income – Conflict affected
		See relevant country list as per World Bank Region
Study design		– Systematic reviews – RCT – Quasiexperimental study – Case‐control – Cohort – Controlled trial
Population		– Children – Adults – Elderly – Women – Men – LGBT community – Conflict affected – Disadvantaged – Migrants/refugees – Ethnic minorities
Type of Impairment		– Physical impairment – Visual impairment – Mental impairment – Hearing impairment – Intellectual/learning impairment
Intervention		– Health ○ Promotion ○ Prevention ○ Medical care ○ Rehabilitation ○ Assistive devices – Education ○ Early child development ○ Nonformal ○ Primary and secondary ○ Lifelong learning – Livelihood Skills development Self‐employment Waged employment Financial services Social protection – Social ○ Relationship, marriage and family ○ Personal assistance ○ Culture, religion and arts ○ Sports, recreation and leisure ○ Access to justice – Empowerment ○ Social mobilisation ○ Political Participation ○ Language and communication ○ Self‐help groups and Disabled People's Organisation – Advocacy and Governance ○ Advocacy and Governance
Outcome		– Health ○ Mental health and cognitive development ○ Access to health services ○ Immunization ○ Health check‐up ○ Rehabilitation ○ Access to assistive devices ○ Nutrition ○ Morbidity and mortality – Education ○ Enrolment to primary, secondary and tertiary education ○ Attendance ○ Education in mainstream education facilities/inclusive education ○ Social and life skill development ○ Access to educational services – Livelihood ○ Employment in formal and informal sector ○ Access to job market ○ Control over own money ○ Access to financial services such as grants and loans ○ Poverty and out‐of‐pocket payment ○ Access to social protection programs ○ Participation in development of inclusive policies – Social ○ Stigma and discrimination ○ Safety ○ Participation in mainstream recreational, leisure and sports activity ○ legal rights ○ Access to justice ○ Participation in cultural and religious activity ○ Interpersonal interaction and relationships ○ Social identity and responsibilities – Empowerment ○ Informed choices ○ Positions in public institutions and Judiciary ○ Voting rights ○ Representation at community level ○ Advocacy

## APPENDIX D

**Table D1:** Search strategy

Search string/key words (For ovid medline platform)
Developing Country Free Text
– (developing OR less‐developed OR less* developed OR "under developed" OR underdeveloped OR under‐developed OR middle‐income OR "middle income" OR "low income" OR low‐income OR underserved OR "under served" OR deprived or poor*) adj3 (countr* OR nation OR population OR world OR state OR economy OR economies).mp – ("third world" OR L&MIC OR L&MIC OR LAMIC OR LDC OR LIC OR LMIC* OR lami countr* OR transitional countr*).mp – (Africa OR "Sub‐Saharan Africa" OR "North Africa" OR "West Africa" OR "East Africa" OR Algeria OR Angola OR Benin OR Botswana OR Burkina Faso OR Burundi OR Cameroon OR "Cape Verde" OR "Central African Republic" OR Chad OR "Democratic Republic of the Congo" OR "Republic of the Congo" OR Congo OR "Cote d'Ivoire" OR "Ivory Coast" OR Djibouti OR Egypt OR "Equatorial Guinea" OR Eritrea OR Ethiopia OR Gabon OR Gambia OR Ghana OR Guinea OR Guinea‐Bissau OR Kenya OR Lesotho OR Liberia OR Libya OR Madagascar OR Malawi OR Mali OR Mauritania OR Morocco OR Mozambique OR Namibia OR Niger OR Nigeria OR Rwanda OR "Sao Tome" OR Principe OR Senegal OR "Sierra Leone" OR Somalia OR Somaliland OR "South Africa" OR "South Sudan" OR Sudan OR Swaziland OR Tanzania OR Togo OR Tunisia OR Uganda OR Zambia OR Zimbabwe).mp. – ("South America" OR "Latin America" OR "Central America" OR Mexico OR Argentina OR Bolivia OR Brazil OR Chile OR Colombia OR Ecuador OR Guyana OR Paraguay OR Peru OR Suriname OR Uruguay OR Venezuela OR Belize OR "Costa Rica" OR "El Salvador" OR Guatemala OR Honduras OR Nicaragua OR Panama).mp. – ("Middle East" OR "South‐East Asia" OR "Indian Ocean Island*" OR "South Asia" OR "Central Asia" OR Caucasus OR Afghanistan OR Azerbaijan OR Bangladesh OR Bhutan OR Burma OR Cambodia OR China OR Georgia OR India OR Iran OR Iraq OR Jordan OR Kazakhstan OR Korea OR "Kyrgyz Republic" OR Kyrgyzstan OR Lao OR Laos OR Lebanon OR Macao OR Mongolia OR Myanmar OR Nepal OR Oman OR Pakistan OR Russia OR "Russian Federation" OR "Saudi Arabia" OR Bahrain OR Indonesia OR Malaysia OR Philippines OR Sri Lanka OR Syria OR "Syrian Arab Republic" OR Tajikistan OR Thailand OR Timor‐Leste OR Timor OR Turkey OR Turkmenistan OR Uzbekistan OR Vietnam OR "West Bank" OR Gaza OR Yemen OR Comoros OR Maldives OR Mauritius OR Seychelles).mp. – ("Pacific Islands" OR "American Samoa" OR Fiji OR Guam OR Kiribati OR "Marshall Islands" OR Micronesia OR New Caledonia OR "Northern Mariana Islands" OR Palau OR "Papua New Guinea" OR Samoa OR "Solomon Islands" OR Tonga OR Tuvalu OR Vanuatu).mp
Systematic review key words
– ((systematic* or synthes*) adj3 (research or evaluation* or finding* or thematic* or report or descriptive or explanatory or narrative or meta* or review* or data or literature or studies or evidence or map or quantitative or study or studies or paper or impact or impacts or effect* or compar*)).ti,ab,sh.
OR
("meta regression" or "meta synth*" or "meta‐synth*" or "meta analy*" or "metaanaly*" or "meta‐analy*" or "metanaly*" or "metaregression" or "metaregression" or "methodologic* overview" or "pool* analys*" or "pool* data" or "quantitative* overview" or "research integration").ti,ab,sh.
OR
(review adj3 (effectiveness or effects or systemat* or synth* or integrat* or map* or methodologic* or quantitative or evidence or literature)).ti,ab,sh.
Qualitative review search term
((("meta ethnography" OR "meta ethnographic") OR ("meta synthesis") OR (synthesis AND ("qualitative literature" OR "qualitative research")) OR ("critical interpretive synthesis") OR ("systematic review" AND ("qualitative research" OR "qualitative literature" OR "qualitative studies")) OR ("thematic synthesis" OR "framework synthesis") OR ("realist review" OR "realist synthesis") OR ((("qualitative systematic review" OR "qualitative evidence synthesis")) OR ("qualitative systematic reviews" OR "qualitative evidence syntheses")) OR (("quality assessment" OR "critical appraisal") AND ("qualitative research" OR "qualitative literature" OR "qualitative studies")) OR (("literature search" OR "literature searching" OR "literature searches") AND ("qualitative research" OR "qualitative literature" OR "qualitative studies")) OR (Noblit AND Hare)) OR ("meta narrative" OR "meta narratives" OR "narrative synthesis")
Disability key words
– ((Disable* or Disabilit* or Handicapped) adj5 (person* or people or child*or adolescen* or women or mother*or maternal, group)).sh,ti,ab. – ((physical* or intellectual* or learning or psychiatric* or sensory or motor or neuromotor or cognitive or mental* or developmental or communication or learning) adj2 (disabilit* or disabl* or handicap*)).ti,ab – ((cognitive* or learning or mobility or sensory or visual* or vision or sight or hearing or physical* or mental* or intellectual*) adj2 impair*).ti,ab – ((mental health or mental disorder* or depress* or anxiety or psychiat* or well‐being or quality of life or self‐esteem or self perception)). ti,ab – ((mental* or emotional* or psychiatric or neurological or neurologic) adj2 (disorder* or ill or illness*)).ti,ab (deaf or deafness or blind or blindness).ti,ab – exp Disabled persons/ – (Autis* or Dyslexi* or Down* Syndrome or Mongolism or Trisomy 21).sh,ti,ab. – exp Intellectual disability/or exp Developmental Disabilities/or exp Child Development Disorders, Pervasive/or exp Communication Disorders/ – ((Intellectual* or Educational*or Mental* or Psychological* or Developmental) adj5 (impair* or retard* or deficienc* or Deficien* or disable* or disabili* or handicap* or ill*)).sh,ti,ab. – ((Hearing or Acoustic or Ear*) adj5 (loss* or impair* or deficienc* or disable* or disabili* or handicap*)).sh,ti,ab. – ((Visual* or Vision or Eye*) adj5 (loss* or impair* or deficienc* or disable* or disabili* or handicap*)).sh,ti,ab. – (Deaf* or Blind*).sh,ti,ab – exp Cerebral palsy/or exp Spina Bifida Cystica/or exp Spina Bifida Occulta/or exp Muscular dystrophies/or exp Arthritis/or exp Osteogenesis Imperfecta/or exp Musculoskeletal Abnormalities/or exp Brain Injuries/or exp Amputation/or exp Clubfoot/or exp Poliomyelitis/or exp Paraplegia/or exp Hemiplegia/or exp Stroke/ – (Cerebral pals* or Spina bifida or Muscular dystroph* or Arthriti* or Osteogenesis imperfecta or Musculoskeletal abnormalit* or Musculo‐skeletal abnormalit* or Muscular abnormalit* or Skeletal abnormalit* or Limb abnormalit* or Brain injur* or Amputation* or Clubfoot or Poliomyeliti* or Paraplegi* or Paralys* or Paralyz* or Hemiplegi* or Stroke* or Cerebrovascular accident*).sh,ti,ab. – (Physical* adj5 (impair* or deficienc* or disable* or disabili* or handicap*)).sh,ti,ab.
**Equity terms:**
– ((social* or socio‐economic or socioeconomic or economic or structural or material) adj3 (advantage* or disadvantage* or exclude* or exclusion or include* or inclusion or status or position or gradient* or hierarch* **or class* or determinant*)).sh,ti,ab.** – (equit* or inequit* or inequ**alit* or disparit* or equality).sf,ti,ab.**
